# Fluorescent Carbon Dioxide-Based Polycarbonates Probe for Rapid Detection of Aniline in the Environment and Its Biomarkers in Urine

**DOI:** 10.3390/polym16040541

**Published:** 2024-02-17

**Authors:** Yun Liu, Wen-Zhen Wang, Zhi-Ping Zhang, Chun-Bao Du, Lei-Lei Li, Chen Zhao, Hong-Jiu Li, Qing Huang

**Affiliations:** Shaanxi Engineering Research Center of Green Low-Carbon Energy Materials and Processes, College of Chemistry and Chemical Engineering, Xi’an Shiyou University, Xi’an 710065, China; 22211071037@stumail.xsyu.edu.cn (Y.L.); zhipingzhang@xsyu.edu.cn (Z.-P.Z.); duchunbao@xsyu.edu.cn (C.-B.D.); lll@xsyu.edu.cn (L.-L.L.); 20211070835@stumail.xsyu.edu.cn (C.Z.); 21211070915@stumail.xsyu.edu.cn (H.-J.L.); 21211070884@stumail.xsyu.edu.cn (Q.H.)

**Keywords:** carbon dioxide, functional material, polycarbonate, fluorescent probe, aniline, para-amino phenol

## Abstract

Aniline compounds, as a class of widely used but highly toxic chemical raw materials, are increasingly being released and accumulated in the environment, posing serious threats to environmental safety and human health. Therefore, developing detection methods for aniline compounds is of particular significance. Herein, we synthesized the fluorescent third monomer cyano-stilbene epoxide M and ternary copolymerized it with carbon dioxide (CO_2_) and propylene oxide (PO) to synthesize carbon dioxide-based polycarbonate (PPCM) with fluorescence recognition functions, as well as excellent performance, for the first time. The results revealed that the PPCM fluorescent probe exhibited typical aggregation-induced luminescence properties and could be quenched by aniline compounds. The probe presented anti-interference-specific selectivity for aniline compounds, and the detection limit was 1.69 × 10^−4^ M. Moreover, it was found to be a highly sensitive aniline detection probe. At the same time, the aniline biomarker p-aminophenol in urine could also be detected, which could expand the potential applications of polymers in the fluorescence-sensing field.

## 1. Introduction

With the rapid developments in modern industry, the excessive use of fossil fuels has resulted in a continuous increase in carbon dioxide (CO_2_) emissions, leading to serious greenhouse effects and environmental pollution [1,2]. Currently, CO_2_ management is primarily divided into two categories: one involves reducing the production of CO_2_ from the source, while the other involves capturing and utilizing CO_2_ that is already produced [3,4]. From the perspective of such CO_2_ resource utilization, CO_2_ is not only a greenhouse gas but also a low-priced and widely available renewable carbon resource [5,6,7]. Such CO_2_ utilization can not only reduce its concentration in the environment but also aid in the production of energy products, chemicals, and materials, thus alleviating environmental pressure while simultaneously reaping economic benefits. Accordingly, some methods have been developed for the chemical conversion of CO_2_; these include the electrochemical method [8], the thermal catalytic method [9], and the photocatalytic method [10], which can convert CO_2_ into methanol [11], formic acid [12], dimethyl carbonate [13], polyurethane [14], polyether ester polyol [15], polycarbonate [16], and other high value-added chemicals. Among these, poly(propylene carbonate) [17] (PPC) prepared through the copolymerization of CO_2_ and propylene oxide (PO) is a biodegradable polymer with a good oxygen barrier, transparency, and biocompatibility. It is widely used in food packaging [18], film materials [19], barrier materials [20], biomedical materials [21], and other fields. Despite these advantages, PPC still has some defects. On the one hand, PPC synthesized by this pathway is an amorphous polymer with flexible chain structures, low glass transition temperatures [22], poor thermal stability, and inferior mechanical properties. On the other hand, the limited availability of functional groups that can be modified by PO complicates the preparation of functional PPC with special uses. Therefore, PPC modification has emerged as an urgent problem [23,24,25]. Currently, PPC modification methods primarily include physical and chemical methods. Among these, in the physical methods, PPC is modified by blending it with other polymers or inorganic substances, and the methods are mainly divided into solution [26] and melt blending [27]. Although physical methods can more easily improve the thermal stability as well as the mechanical properties of PPC, issues such as poor compatibility and phase separation are often encountered between the blends, and structurally changing the polymer properties is not feasible. Chemical methods involve the selective introduction of appropriate units into the polymer chain and the modulation of the molecular chain structure of the polymer through the chemical reaction copolymerization to obtain polymerization products with more sophisticated stereo and regionally controlled structures. The primary chemical methods used to modify PPC include terpolymerization [28], block copolymerization [29], crosslinking [30], chain transfer [31], and graft copolymerization reactions [32]. Among these, terpolymerization is currently the main method adopted in the study of modified PPC, owing to its flexible third monomer selectivity. In this case, the introduction of a third monomer can not only selectively adjust the structure of PPC and improve its performance but also endow it with unique functions. However, current studies primarily focus on the adhesive, flame-retardant, hydrophilic, and hydrophobic properties [33,34,35,36] of PPC, while studies on PPC with fluorescent properties are rare. This is because PPC synthesized from CO_2_ and PO lacks modifiable functional groups for the introduction of luminescent groups. Therefore, the introduction of luminescent groups is of great research significance for the preparation of PPC with fluorescent properties.

To date, several studies have reported the introduction of luminescent groups into polymers to make them fluorescent, and scientists have discovered that a great variety of fluorescent polymers can be synthesized by introducing different luminescent groups with fluorescent properties into polymer materials. In 2016, Ma et al. [37] polymerized the luminescent moiety tetrastyrene with N-isopropylacrylamide (NIPAM) to prepare a fluorescent polymer material with a low critical eutectic melting temperature (LCST) of 37.5 °C. Owing to the temperature-sensitive properties of the material itself, a sol–gel phase transfer could be observed upon changes near the LCST, which induced a fluorescent response. In 2020, Lenora et al. [38] introduced benzimidazole-based luminescent groups to synthesize a 2,6-bis(2-benzimidazolyl)pyridine-conjugated sesquisiloxane-based semi-branched polymer and investigated its photophysical properties and metal-sensitizing properties. The polymer was found to emit a blue color in both solid and solution states and was expected to be used as a Zn(II) fluorescent sensor. In 2021, Zeng et al. [39] synthesized a coumarin@GDP-Tb dual-emission fluorescent polymer by introducing coumarin-like luminescent groups. These coumarin@GDP-Tb polymers were then found to not only be rich in metal-binding sites but also to have moderate ion-binding power, which made them suitable as fluorescent probes that could be used for S^2−^ detection. Thus, the emergence of numerous fluorescent polymers containing different luminescent groups provides new ideas and good support for the preparation of PPCs with fluorescent properties.

Luminescent groups currently used in polymers include stilbenes [40], tetrastilbenes [41], benzimidazoles [42], rhodamine [43], fluoresceins [44], and coumarins [45]. Among these, cyanostilbene compounds, as a typical class of aggregation-induced emission (AIE) molecules, have not only significant optical properties but also possess simple synthesis routes and allow easy modifications of structures. They have been extensively utilized in various application fields such as detection sensors, organic photoelectric materials, and solar cells [46,47,48]. To prepare PPCs with fluorescent properties, herein, cyanostilbene epoxy compound M was synthesized using the cyanostilbene group [49] as the luminescent group, and PPCM with fluorescent properties was prepared through the copolymerization of cyanostilbene epoxy compound M with CO_2_ and PO for the first time. Following this, the structure and properties of PPCM were characterized and analyzed. The results revealed that PPCM not only had good thermal and mechanical properties but also demonstrated stable luminescence performance in water with aggregation-induced luminescence effects. In addition, PPCM has been previously demonstrated to show good selectivity and anti-interference specificity for aniline [50,51] compounds among several organic compounds while also being capable of detecting aniline pollutants in different environmental water media with high sensitivity and detecting p-aminophenol in urine. It is a novel polymer fluorescent probe that can be used to detect aniline in the environment and aniline biomarkers in urine.

## 2. Experimental

### 2.1. Materials and Methods

PO (99%) was purchased from China, Shanghai Aladdin Co., Ltd. (Shanghai, China) and purified using calcium hydride reflux for 8 h under a nitrogen atmosphere before use. Cobalt acetate tetrahydrate was vacuum-dried at 140 °C to remove crystal water, and toluene was distilled with sodium metal before use. The purified reagents were stored in a dryer containing 4A molecular sieves for later use. CO_2_ (99.99%) was purchased from China, Xi’an Tenglong Chemical Co., Ltd. (Xi’an, China). All other reagents were of analytical grade and used without further purification.

Fourier-transform infrared (FT-IR) spectrometry (Nicolet 6700; Thermo Scientific, Waltham, MA, USA) with attenuated total reflection accessories was used to characterize the structure of the product. Proton nuclear magnetic resonance (^1^H NMR) spectra were obtained (400 M, Bruker, Karlsruhe, Germany) using deuterochloroform as the solvent. The polymers’ molecular weights (M_w_) and the molecular weight distributions (PDI) were measured using gel chromatography (PL-GPC 50, Agilent, Santa Clara, CA, USA) with tetrahydrofuran as an eluent. The glass transition temperature (T_g_) was determined using a DSC-3 differential scanning calorimeter (DSC) under a nitrogen atmosphere at a heating rate of 10 °C/min from 25 to 110 °C. Thermogravimetric testing (TGA) was performed using a thermogravimetric analyzer (TGA/DSC-1) under a nitrogen atmosphere at a heating rate of 10 °C/min from 25 to 500 °C. Each polymer sample was prepared in a dumbbell-shaped strip of 25 mm × 4 mm × 1 mm (length × breadth × thickness) using a universal material testing machine (CMT) and following the ASTM E-104 standard; subsequently, the tensile properties were tested under 50% ± 5% humidity at 25 °C at a tensile speed of 50 mm/min. A Hitachi F-7000 spectrometer equipped with a quartz cuvette of 1 cm optical range and a xenon lamp as the excitation source was used for fluorescence spectroscopy, with excitation and emission slit widths of 10.0 nm.

### 2.2. Synthesis of Monomer M

**Scheme 1:**
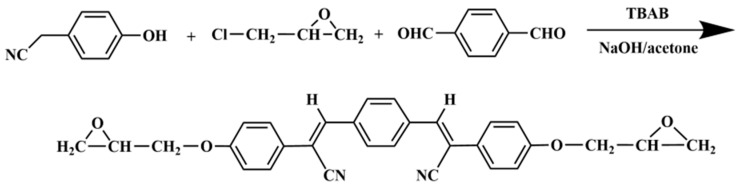
Synthesis route of monomer M.

P-hydroxybenzeneacetonitrile (1.33 g, 0.01 mol) and epichlorohydrin (9.25 g, 0.10 mol) were added to a 250 mL single-necked flask with a magnet and heated to 68 °C. After the ingredients were dissolved, tetrabutylammonium bromide (0.322 g, 0.001 mol) was added; after approximately 8 h of reaction, terephthalaldehyde (0.6000 g, 0.0045 mol) and 150 mL of anhydrous ethanol were added, followed by solid sodium hydroxide (1.20 g, 0.03 mol). The reaction continued for approximately 4–6 h before stopping. After the reaction system had cooled to room temperature, filtration was performed. The solid product was collected, washed with water, and dried to obtain the crude product, which was recrystallized with a mixed solvent of N, N-Dimethylformamide (DMF) and ethanol (*v*:*v* = 1:1) to obtain 1 g of a yellow-green product, i.e., the ((2Z,2′Z)-3,3′-(1,4-phenylene)bis(2-(4-(oxiranyl-2-methoxy)phenyl)acrylonitrile)- epoxide compound, which is referred to as M, as shown in Scheme 1.

### 2.3. Terpolymerization of CO_2_, PO, and M

**Scheme 2:**
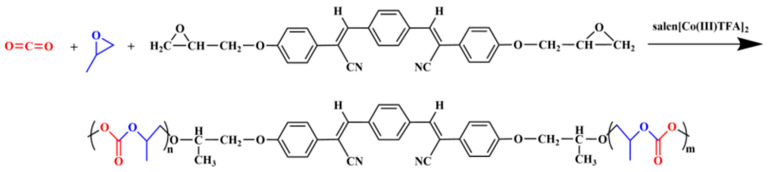
Synthesis route of PPCM.

The terpolymerization of CO_2_, PO, and M is shown in Scheme 2. The novel catalyst dinuclear cobalt-coordinated salen[Co(III)TFA]^2^ (0.05 g) synthesized previously by our group [52] and a certain proportion of M were added into a 250 mL autoclave reactor equipped with a magnetic stirrer and dried for 2 h at 80 °C under vacuum. The autoclave was carefully purged with nitrogen. Subsequently, 11.6 g PO was injected into the autoclave, which was then filled with CO_2_ at 3 MPa. The reaction was carried out at 70 °C for 24 h. Afterward, the reactants were then cooled to room temperature and the pressure was released. The obtained viscous mixture was washed with an anhydrous ethanol solution containing 5% HCl, and the crude product was dissolved in an appropriate amount of CH_2_Cl_2_ and filtered to remove the catalyst. Finally, methanol was precipitated, centrifuged, collected, and dried under a vacuum until a constant weight was achieved. The polymer yield was then calculated.

## 3. Results and Discussion

### 3.1. Monomer Structure Characterization

**Figure 1:**
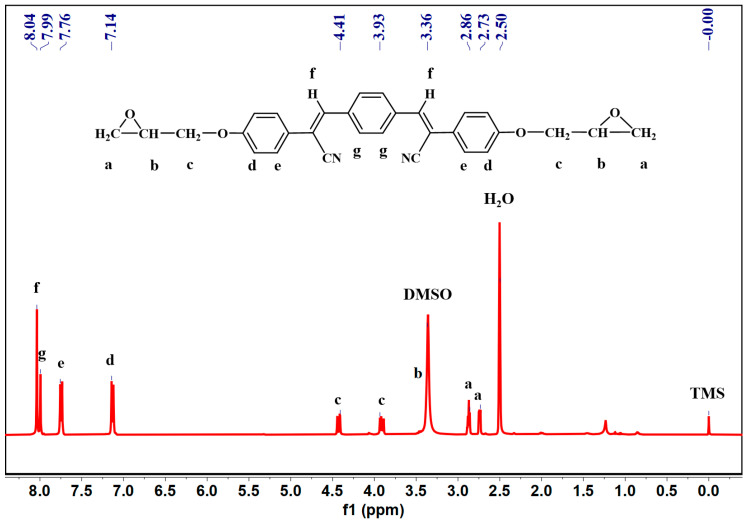
^1^H NMR spectrum of monomer M.

The ^1^H NMR spectrum of the prepared M is shown in Figure 1. The signals at 2.73, 2.86, and 3.56 ppm were assigned to the hydrogen in CH_2_ and CH on the ring in epichlorohydrin, whereas the signals at 3.93 and 3.41 ppm were assigned to the hydrogen in CH_2_ on the side chain in epichlorohydrin. Further, the signals at 7.76–8.04 ppm were attributed to the benzene ring, and the signal at 8.04 ppm was attributed to the hydrogen in the unsaturated double bond. We confirmed that the monomer was successfully synthesized.

### 3.2. CO_2_ Terpolymerization Reaction

Feed ratio is the main factor affecting the copolymerization reaction. Five groups with different feed ratios of PO and M were used to explore the effect of feed ratio on the ternary copolymerization reaction. In the presence of aggregation-induced luminescent monomer M, the ternary copolymerization of CO_2_, PO, and M can be efficiently catalyzed using the dinuclear cobalt complex salen [Co(III)TFA]_2_, and polycarbonate PPCM can be successfully prepared. With the increase in monomer M content in the reaction system, the M_n_ of the copolymer increased from 6352 to 14,577 g mol^−1^ and then gradually decreased to 7335 g mol^−1^; similarly, the yield of the polymer also first increased and then decreased (Table 1). This is because PO, which has been ring-opened and has activity, is more likely to bind to the third monomer M at a higher concentration in the system with the increase in M content in the reaction substrate, and it can quickly link to the molecular chain to obtain a polymer with a high molecular weight. However, when more M monomers are introduced, the relative concentration of PO decreases and the number of active centers decreases, which is not conducive to the polymerization reaction [53]. At this point, the reaction attains equilibrium. In summary, the M_n_ of PPCM was controllable in the range between 6352 and 14,577 g mol^−1^. The yield and molecular weight of the polymer increased with the addition of an appropriate amount of monomer M, and the optimal feed ratio was PO/M = 100:3.

### 3.3. Confirmation of the Copolymer Structure

**Figure 2:**
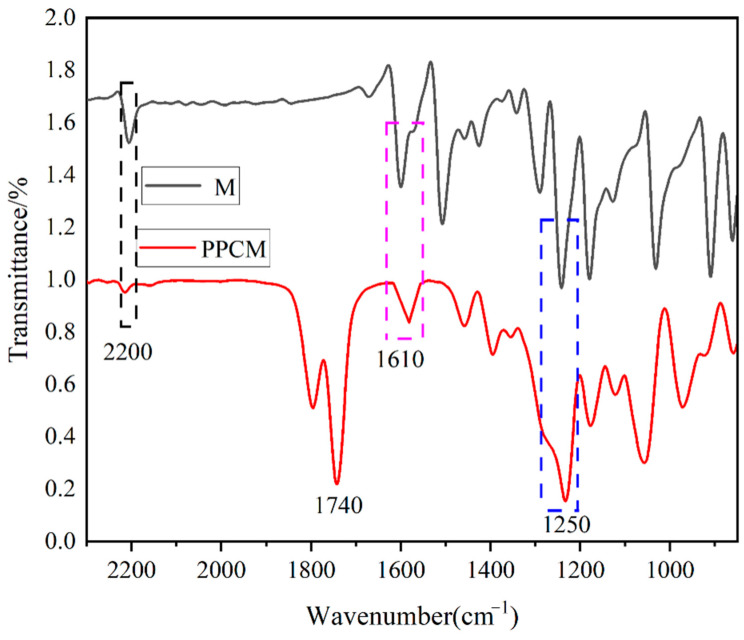
FT-IR spectrum of polymers M and PPCM.

The polymer’s structure was characterized using FT-IR spectroscopy and ^1^H NMR. The FT-IR spectra of monomer M and PPCM are shown in Figure 2. Evidently, the characteristic absorption peak of the cyano group appeared at 2200 cm^−1^ in both the M and PPCM spectra. The peaks at 1250 and 1120 cm^−1^ were the characteristic absorptions of the aromatic ether C-O-C, and the characteristic absorption peak of the double bond near 1610 cm^−1^ was retained, thus indicating that the carbon–carbon double bond did not participate in the polymerization reaction. Additionally, the difference between the PPCM and M spectra is that the C=O stretching vibration of the ester bond appeared near 1740 cm^−1^, thus indicating that the epoxy bond of monomer M was broken into an ester bond of the carbonate chain during the polymerization process. Synthesis of the ternary polymerization product was initially judged to be successful.

**Figure 3:**
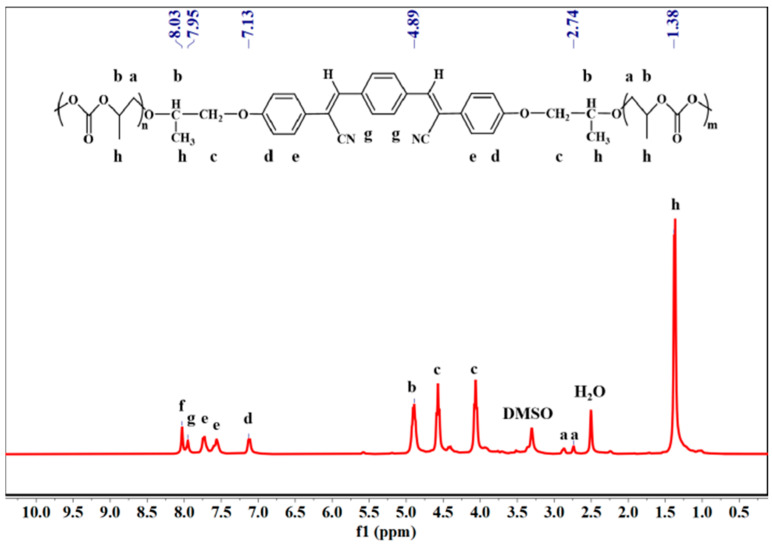
^1^H NMR spectrum of PPCM.

The ^1^H NMR spectrum of PPCM is shown in Figure 3. The signals at 1.38, 2.74, and 4.89 ppm were assigned to the hydrogen atoms in CH_3_, CH_2_, and CH in the carbonate unit of PPC, respectively. The signals at 7.13–7.93 ppm were assigned to the hydrogen in the benzene ring of the M monomer. The signal at 8.03 ppm was attributed to hydrogen in the unsaturated double bond in the M monomer. These results demonstrate the successful formation of the PPCM polymers.

### 3.4. Copolymer Properties

#### 3.4.1. Thermal Properties

**Figure 4:**
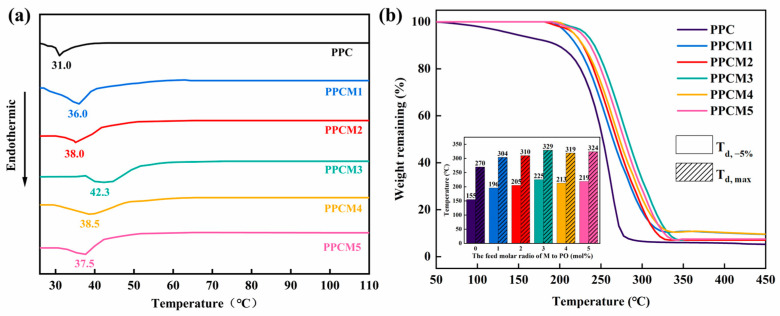
(**a**) DSC curves of PPC and PPCMs; (**b**) TGA curves of PPC and PPCMs.

Thermal properties significantly affect the processing and application of polymer materials. Figure 4a shows the DSC curves of PPC and PPCM copolymers with different feed ratios. Evidently, the T_g_ of the PPCMs was higher than that of PPC. In particular, the T_g_ of PPCM3 was 42.3 °C, which is 36% higher than that of PPC (31.0 °C). This is because introducing a third monomer containing a benzene ring structure changes the chain structure and effectively improves the thermal stability of PPC [54]. Figure 4b shows the TGA curves of the polymers. PPC decomposes at 155 °C, and the maximum thermal decomposition temperature (T_d, max_) is 270 °C. After adding the third monomer M, the T_d, −5%_ and T_d, max_ of PPCM3 could reach 225 and 329 °C, which are 45% and 22% higher than that of PPC, respectively, which is a breakthrough improvement. This improvement in thermal stability was attributed to the incorporation of a rigid third monomer with double bonds, redistribution of the main chain structure of the molecular chain, and improvement in the stiffness of the polymer. This broadens the thermal processing temperature range of the copolymers, thereby enabling a more comprehensive range of applications.

#### 3.4.2. Mechanical Properties of the Polymer

**Figure 5:**
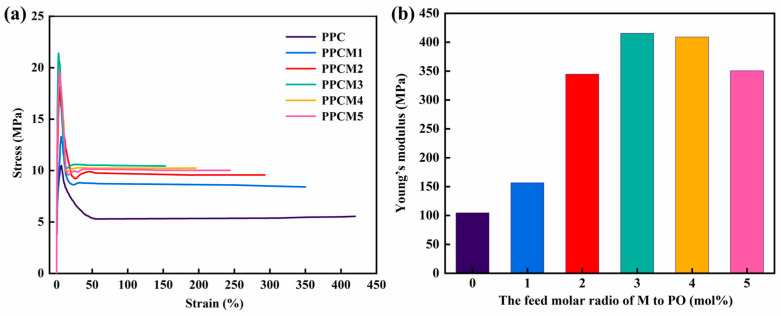
(**a**) Strain–stress curves of PPC and PPCM_S_; (**b**) Young’s modulus of PPC and PPCMs.

The main chain of PPC comprises ester bonds and a small number of ether bonds; further, no strong forces exist between the molecules. The molecular chain slides easily, and its mechanical strength is low [55]. Therefore, the PPC must be modified mechanically. The mechanical properties of PPC and PPCMs are shown in Figure 5. Evidently, the tensile strength and Young’s modulus of the PPCMs are higher than those of pure PPC, and their elongation at break is lower than that of pure PPC. In particular, the maximum tensile strength of PPCM3 was 21.4 MPa, and Young’s modulus was 415.3 MPa, which is nearly four times that of PPC, and its elongation at break was only 153%. The significant improvement in the mechanical properties of PPCM is closely related to the introduction of a third monomer, M, which is attributed to the rigid benzene ring group in M and its large steric hindrance. The introduction of M changes the main chain structure and limits the chain movement of PPC, thus improving the rigidity of the main chain of PPC. PPCM is not only stable at high temperatures but can also withstand greater pressures, further expanding its industrial application range.

#### 3.4.3. Aggregation-Induced Luminescence Properties of the Polymer

**Figure 6:**
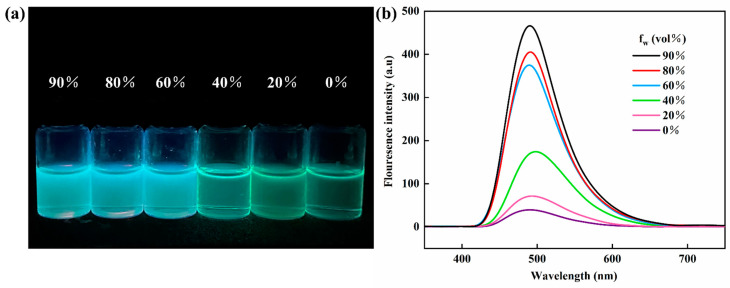
(**a**) Photoluminescence spectra of PPCM (M_n_ = 14,577 g mol^−1^) in the mixed solution of THF and H_2_O (concentration: 1 mg mL^−1^, λ_ex_ = 365 nm); (**b**) fluorescence plot of PPCM in a mixed solution of THF and H_2_O under UV light (365 nm).

Reportedly, cyanostilbene derivatives are typical aggregation-induced luminescent molecules that emit weak or even no light in dilute solutions, whereas the aggregated state or solid film state produces intense fluorescence. Considering the mutual solubility of good and poor solvents, tetrahydrofuran was selected as the good solvent, while water was selected as the poor solvent. Polycarbonate PPCM was dissolved in a mixed solvent of THF and H_2_O (the volume ratios of H_2_O:THF were 9:1 (corresponding to 90% in the graph), 8:2 (corresponding to 80% in the graph), 6:4 (corresponding to 60% in the graph), 4:6 (corresponding to 40% in the graph), 2:8 (corresponding to 20% in the graph) or 0:10 (corresponding to 0% in the graph)). Figure 6 shows the photoluminescence spectra of PPCM in a mixture of THF and H_2_O for various volume ratios. The fluorescence intensity of the polymer increases with an increase in the content of poor solvent water, thus indicating AIE properties. Owing to the poor solubility of PPCM in water, the system demonstrated aggregated behavior, and the free rotational movement of the benzene ring was restricted; that is, intramolecular rotation was blocked, causing the compound molecules to transition from the distorted conformation to the near-planar conformation, thus increasing the effective conjugation length of the molecules and gradually enhancing the fluorescence luminescence intensity [56]. This shows that PPCM synthesized through copolymerization with CO_2_ as the raw material still exhibits aggregation-induced luminescence properties.

### 3.5. Applications of Copolymer Probes in the Environment

#### 3.5.1. Recognition of Aniline Compounds via Copolymer PPCM

**Figure 7:**
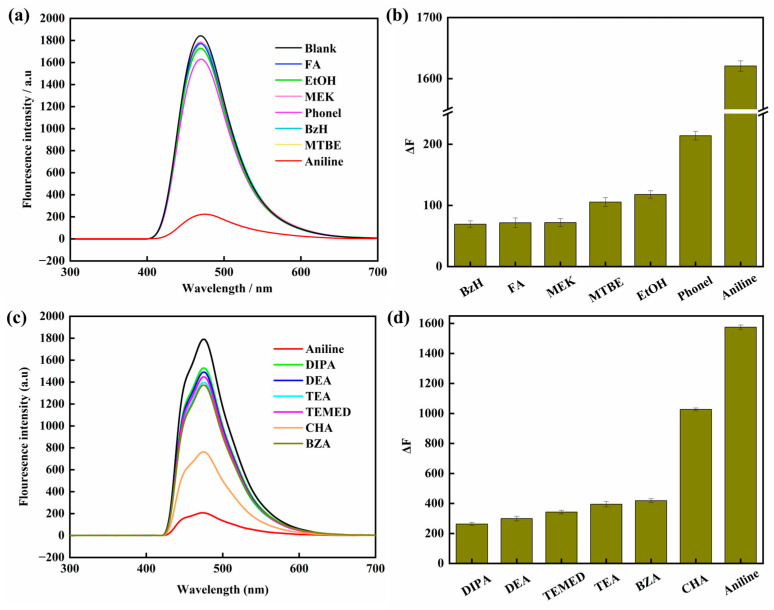
(**a**) Changes in fluorescence spectra of PPCM solution after the addition of different organic compounds; (**b**) changes in fluorescence intensity of PPCM solution after the addition of different organic compounds; (**c**) changes in fluorescence spectra of PPCM solution after the addition of different amines; (**d**) vhanges in fluorescence intensity of PPCM solution after the addition of different amines.

In order to investigate the recognition function of copolymer PPCM on specific organic compounds, several common organic compounds (formic acid (FA), ethanol (EtOH), butanone (MEK), phenol, benzaldehyde (BzH), methyl tert-butyl ether (MTBE), aniline) were selected for comparison, and 2 mL of 2 × 10^−4^ g/mL PPCM solution (THF/H_2_O = 1:9, *v*/*v*) was added to the fluorescence cuvette, after which 20 μL of different organic compounds, and the changes in the fluorescence intensity of the solutions were tested via fluorescence spectroscopy. As can be seen in Figure 7, the fluorescence intensity did not change much when formic acid, ethanol, butanone, phenol, benzaldehyde, and methyl tert-butyl ether were added. When aniline was added, the fluorescence intensity decreased a lot. In order to further investigate the identification of aniline via the copolymer PPCM, different amine compounds (diisopropylamine (DIPA), diethylamine (DEA), triethylamine (TEA), tetramethyldiethylamine (TEMED), cyclohexylamine (CHA), and benzylamine (BZA)) were selected for comparison. In the fluorescence cuvette, 2 mL of 2 × 10^−4^ g/mL PPCM solution (THF/H_2_O = 1:9, *v*/*v*) was added, and in this, 20 μL solutions of different amines (at a concentration of 1 mol/L) were added, respectively. Changes in the fluorescence intensities of the solutions were measured via fluorescence spectrometry. As can be seen in Figure 7, when DIPA, DEA, TEA, TEMED, and BZA were added, the fluorescence intensities did not change significantly and decreased by approximately 15% in all cases. When CHA was added, the fluorescence intensity decreased by approximately 55%. By contrast, when aniline was added, the fluorescence intensity decreased by approximately 90%, thus exhibiting the best bursting effect. These results suggest that PPCM has high selectivity for aniline and no obvious fluorescence response to other amines.

**Figure 8:**
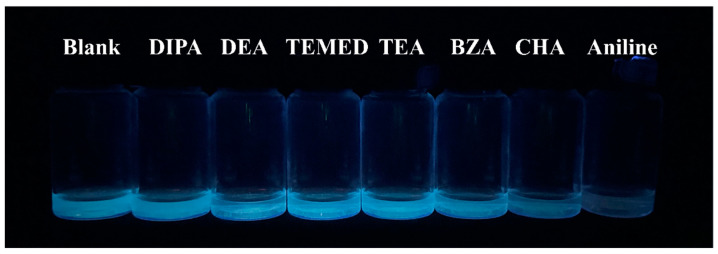
Fluorescence of PPCM solution under UV lamp (365 nm) after addition of different amine compounds.

We also observed comparative plots of the PPCM solution (far left) with the addition of 20 μL, 1 mol/L of amines (aniline, DIPA, DEA, TEMED, BZA, and CHA, from left to right) with the naked eye, under ultraviolet (UV) light irradiation (365 nm) (Figure 8), and the same conclusions were drawn.

To explain the fluorescence sensing of aniline using the PPCM copolymer, we investigated its detection mechanism. Molecular orbital theory focuses on the fact that any electron in a molecule can be viewed as a charged particle focuses on the fact that any electron in a molecule can be viewed as a charged particle moving in a potential field composed of all nuclei and the rest of the electrons. The wavefunction of a single-electron motion state in a molecule is a molecular orbital. The molecular orbital can be obtained by a linear combination of the atomic orbital wave functions in the molecule, and each molecular orbital has a corresponding energy. In molecular orbital theory, the most energetic molecular orbitals are generally referred to as the highest occupied molecular orbitals in the presence of an electron arrangement.

**Figure 9:**
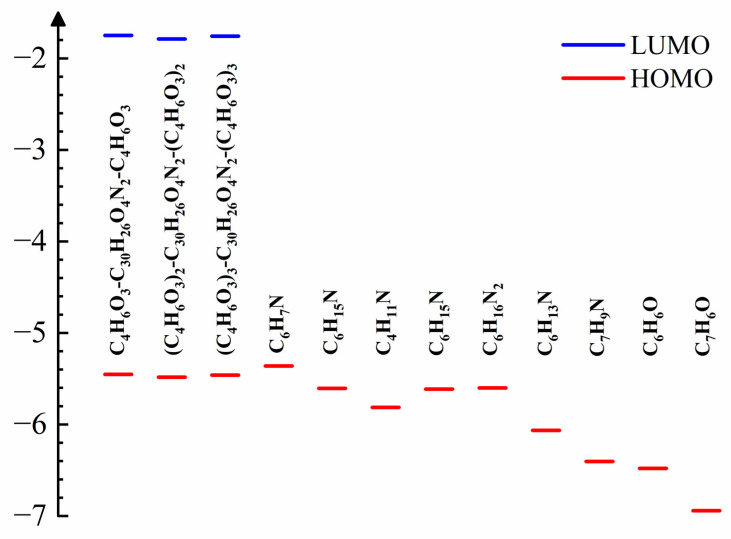
Energy level diagram of PPCM with various types of amines.

This study investigated the structure and electronic properties of molecules and polymers using Gaussian 16 (Revison A.03 WIN64) software based on the density functional theory (DFT) calculation method B3LYP/6–31g (d, p). The energy level diagrams of seven amine compounds (aniline, DIPA, DEA, TEA, TEMED, CHA, and BZA), two aromatic compounds (phenol and BzH), and the polymer PPCM(C_4_H_6_O_3_)_n_-C_30_H_26_O_4_N_2_-(C_4_H_6_O_3_)_m_ (where n and m are taken as 1, 2, or 3) were calculated and obtained. The results are shown in Figure 9. Evidently, the HOMO energy levels of aniline are higher than those of DIPA, DEA, TEA, TEMED, CHA, BZA, phenol and BzH. And, only the HOMO energy level of aniline was higher than that of the polymer PPCM(C_4_H_6_O_3_)_n_-C_30_H_26_O_4_N_2_-(C_4_H_6_O_3_)_m_ (n, m are taken as 1, 2, and 3 respectively). Based on the experimental results, the fluorescence burst of the polymer PPCM was observed only for aniline, and the detection mechanism was hypothesized to be the photoinduced electron transfer [57] (PET) mechanism. The fluorescence quenching can be attributed to the donor–acceptor electron transfer between aniline and the polymer PPCM. When the PPCM system receives a certain amount of energy under light irradiation, the electrons in the highest occupied orbital are excited and move to the lowest unoccupied orbital. Because the HOMO energy level of aniline is higher than that of PPCM, the electrons in the HOMO energy level of aniline move to the HOMO energy level of PPCM, resulting in the fluorescence quenching of the system [58,59].

#### 3.5.2. Anti-Interference Experiments on Aniline Using the Copolymer PPCM

**Figure 10:**
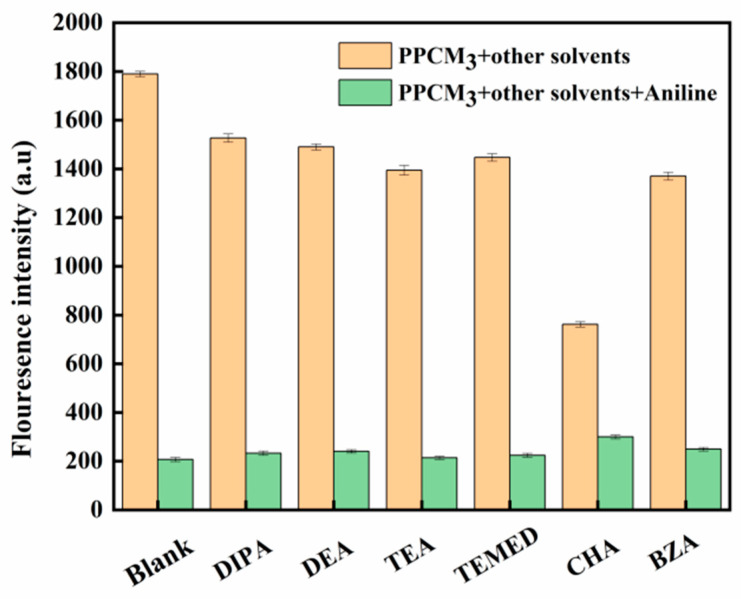
Anti-interference fluorescence intensity plots for different amine compounds.

When detecting the fluorescence recognition of PPCM, we observed the specific selective recognition of aniline and investigated its anti-interference ability during the recognition process. The competitive experiments (Figure 10) suggested that the presence of other amine compounds did not affect the response of aniline, except for the expected complete quenching of aniline. These results indicate that PPCM has anti-interference selectivity toward aniline and can be used as an effective method for detecting aniline.

#### 3.5.3. Sensitivity Testing of the Copolymer PPCM to Aniline

**Figure 11:**
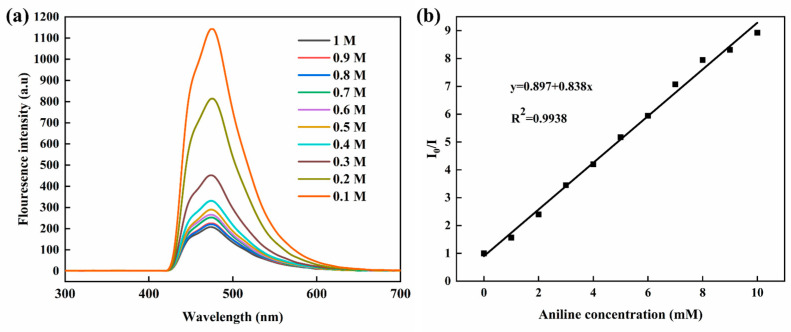
(**a**) Fluorescence spectra of PPCM for various concentrations of aniline; (**b**) linear curve of fluorescence intensity at 475 nm versus aniline concentration.

To explore the sensitivity of the PPCM copolymer to aniline, we investigated the changes in the intensity of its fluorescence spectrum with the addition of different amounts of aniline; PPCM was excited at 365 nm. In a fluorescence cuvette, separate solutions were prepared by adding 2 mL of 2 × 10^−4^ g/mL PPCM solution (THF/H_2_O = 1:9, *v*/*v*) and 20 μL of aniline at different concentrations. Figure 11a shows that the fluorescence intensity in the system decreased significantly with increasing the aniline concentration. When 1 M of aniline was added, the fluorescence intensity decreased to its lowest value. Simultaneously, the Stern–Volmer equation {*I*_0_/*I* = 1 + *K*_sv_[M]} was fitted to this part of the data [60] (Figure 11b). *I*_0_ and *I* represent the initial fluorescence intensity of PPCM at 475 nm and the fluorescence intensity after the addition of aniline, respectively. [M] represents the concentration of aniline, and *K*_sv_ is the quenching constant. As can be seen from Figure 11, the fluorescence intensity of the system showed a linear decrease with the addition of aniline (R^2^ = 0.993, *K*_sv_ = 8.4 × 10^2^ M^−1^), with a larger value of *K*_sv_, which indicates that PPCM has a better sensing sensitivity to aniline.

**Figure 12:**
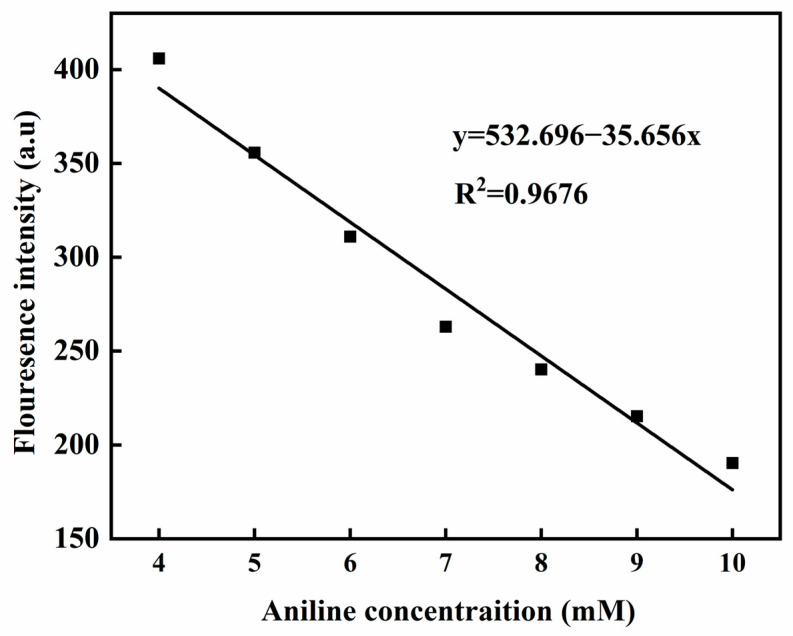
Linear curve of PPCM fluorescence intensity at 475 nm versus aniline concentration.

The primary measure of the sensitivity of a fluorescent chemosensor is the determination of the lowest detection limit of the substance under test; the lower the detection limit, the higher the sensitivity. The detection limit of aniline can be easily calculated from the fluorescence titration data. The fitted curves showed excellent linearity at high concentrations in the aniline concentration range of 0–10 mM (Figure 12).

In addition, the limit of detection (LOD) of PPCM for aniline was calculated using the following two formulae:

$$\sigma =\sqrt{\frac{\sum {\left(F-F_{0}\right)}^{2}}{N-1}}$$

$$LOD=\frac{3\sigma }{k}$$

where σ is the standard deviation of the blank group, *F*_0_ is the fluorescence intensity of the sample at the excitation (475 nm), *F* is the average value of *F*_0_, and *k* is the slope of the relationship curve between the fluorescence intensity at the excitation (475 nm) and the low concentration range of aniline. According to the formula of LOD, the calculated detection limit of PPCM is 1.69 × 10^−4^ M. This demonstrates the suitability of PPCM for detecting environmental aniline with high sensitivity and a low detection limit. The above experiments showed that the PPCM solution had a significant effect on aniline detection and high sensitivity.

#### 3.5.4. Application of Copolymer PPCM in Different Water Samples

To investigate the practical application of copolymer PPCM in the environment, we studied its response to aniline in real environmental water samples (tap water, river water, and wastewater). These water samples were all filtered before use. Two milliliters of the 2 × 10^−4^ g/mL PPCM solution (THF/H_2_O = 1:9, *v*/*v*) was added to a fluorescent cuvette, and this was followed by the addition of 20 μL of aniline solutions with varying concentrations (0.1, 0.5, and 1 M). The standard addition recovery method was adopted to evaluate the feasibility of the PPCM probe for detecting aniline from actual water samples. The corresponding results are summarized in Table 2. The recovery rate of aniline from the different water samples was between 94.2% and 109.0%, indicating that the PPCM probe had high selectivity for aniline in these water samples. Thus, fluorescence detection can be applied to aniline detection in actual water samples.

#### 3.5.5. Recognition of Aniline Compounds via Polymer PPCM

**Figure 13:**
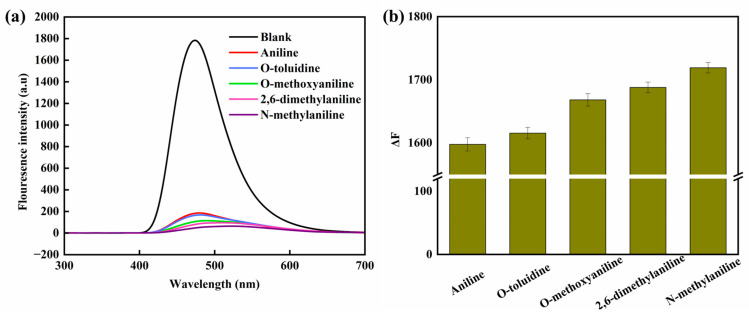
(**a**) Changes in fluorescence spectra of PPCM solution after addition of different aniline analogs. (**b**) Changes in fluorescence intensity of PPCM solution after addition of different aniline analogs.

In addition to aniline, aniline compounds, as important chemical raw materials and intermediates, are also highly toxic, and a small amount can be a poison, which can cause hemolytic anemia, liver damage resulting in toxic liver disease, and carcinogenic, teratogenic, and mutagenic effects. The emission of aniline compounds into the environment is increasing with the development of agriculture and industry, and the damage to the environment and human health is becoming increasingly severe. Therefore, the identification of aniline compounds is of great significance. Five common aniline compounds (aniline, o-toluidine, o-methoxyaniline, 2,6-dimethylaniline, and N-methylaniline) in environmental pollutants were selected to analyze the response effect of the polymer PPCM. Moreover, 2 mL of 2 × 10^−4^ g/mL PPCM solution (THF/H_2_O = 1:9, *v*/*v*) was added to the fluorescence cuvette. Then, 20 μL solutions of aniline, o-toluidine, o-methoxyaniline, 2,6-dimethylaniline and N-methylaniline (all at a concentration of 1 mol/L) were added to form sample solutions for analysis. Changes in the fluorescence intensity of the system were observed via fluorescence spectrometry. Figure 13 shows that the PPCM polymer has a good fluorescence quenching effect on the five aniline compounds. These results broaden the detection range of polymer PPCM in the environment, thus making PPCM more practical and promising for practical detection.

### 3.6. Recognition of Aniline Biomarkers in Urine by Polymer PPCM

**Figure 14:**
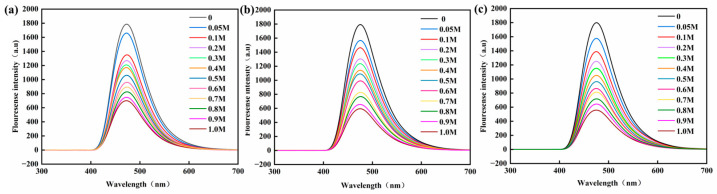
(**a**) Fluorescence spectra of PPCM for various concentrations of p-aminophenol. (**b**) Fluorescence spectra of PPCM for various concentrations of p-aminophenol in artificial urine (pH = 4.7). (**c**) Fluorescence spectra of PPCM for various concentrations of p-aminophenol in artificial urine (pH = 5.7).

Aniline, as a ubiquitous, highly toxic environmental pollutant, can be enriched by inhalation, skin absorption, and the food chain. High concentrations of aniline can lead to toxic methemoglobinemia, causing damage to the human liver, kidney, skin, and other tissues, and is considered to be a potential carcinogen [61]. However, due to individual differences, the concentration level of aniline in the environment cannot accurately and truly reflect its concentration level in the human body. Aniline absorbed by the human body can be metabolized into a biomarker p-aminophenol in the body, which is then excreted with urine [62]. P-aminophenol, a metabolite of aniline in urine, is considered to be an important exposure biomarker for the effective monitoring of aniline. Therefore, the effective detection of p-aminophenol in human urine is of great significance in occupational health. In order to investigate the sensitivity of copolymer PPCM to p-aminophenol, we studied the change in fluorescence spectral intensity under different amounts of p-aminophenol. In a fluorescence cuvette, separate solutions were prepared by adding 2 mL of 2 × 10^−4^ g/mL PPCM solution (THF/H_2_O = 1:9, *v*/*v*) and 20 μL of p-aminophenol at different concentrations. Figure 14a shows that the fluorescence intensity in the system decreased significantly with increasing aniline concentration. When 1 M of aniline was added, the fluorescence intensity decreased to its lowest value. A study was carried out to investigate the practical application of copolymer PPCM in urine. Since the pH of human urine is generally weakly acidic, we studied the response of copolymer PPCM to p-aminophenol in artificial urine (pH = 4.7, pH = 5.7). The results are shown in Figure 14b,c. The probe PPCM has good sensing performance for the metabolite p-aminophenol of aniline in simulated human urine.

## 4. Conclusions

In this study, the ternary copolymerization of CO_2_, PO, and the cyanostilbene-like monomer M produced a fluorescent functional copolymer PPCM. The results revealed that the modified PPCM exhibited superior thermal and mechanical properties. The T_g_ of PPCM reached a maximum of 42.3 °C, and the minimum T_g_ was also higher than that of PPC. The T_d, −5%_ increased from 155 to 225 °C, and the T_d, max_ also increased from 270 to 329 °C. Mechanically, the tensile strength of the copolymer increased from 10.5 to 21.4 MPa with a significant increase in M content. Moreover, after releasing the tensile force, the tensile permanent deformation of the crosslinked material reduced significantly, and the elongation at break reduced from 420% to 153%. The improvement of mechanical properties was distinct. As a novel fluorescent probe with an aggregation-induced luminescence effect, PPCM showed high selectivity and sensitivity for aniline compounds among different compounds, with a detection limit of 1.69 × 10^−4^ M. The application of this fluorescent probe to the detection of aniline compounds in real water samples yielded good spiked recoveries, indicating that this PPCM fluorescent probe has a potential practical application value in the selective identification of aniline compounds. In addition, the polymer PPCM has great potential as a fluorescent sensor for the detection of aniline biomarkers in human urine, which is of great significance to human health.

## Figures and Tables

**Table 1:** Catalytic results of copolymerization of PO and M in different molar ratios ^a^.

Polymer	n[PO]:n[M]	Yield (%)	M_n_ (g/mol) ^b^	PDI ^c^
PPCM1	100:1	32	6352	1.43
PPCM2	100:2	55	8570	1.14
PPCM3	100:3	70	14577	1.27
PPCM4	100:4	68	8530	1.64
PPCM5	100:5	65	7335	1.86

^a^ Polymerization conditions: 0.05 g of dinuclear cobalt complex salen[Co(III)TFA]_2_ catalyst; CO_2_ pressure, 3.0 MPa; 70 °C; 24 h. ^b^ Mn: the average molecular weight (g·mol^−1^) determined via gel permeation chromatography. ^c^ PDI: polydispersity index (M_w_/M_n_); M_w_: average molecular weight (kg·mol^−1^) determined via gel permeation chromatography.

**Table 2:** Analysis of the detection results of aniline in actual water samples via probe PPCM (2 × 10^−4^ g/mL).

Sample	Spiked (M)	Found (M)	Recovery (%)
Tap water	0.10	0.109	109.0
	0.50	0.514	102.8
	1.0	0.963	96.3
River water	0.10	0.104	104.0
	0.50	0.508	101.6
	1.0	1.085	108.5
Waste water	0.10	0.097	97.0
	0.50	0.516	103.2
	1.0	0.942	94.2

## Data Availability

Data are contained within the article.
